# A qualitative exploration of the use of calendar landmarking instruments in cancer symptom research

**DOI:** 10.1186/s12875-014-0167-8

**Published:** 2014-10-25

**Authors:** Katie Mills, Jon Emery, Camilla Cheung, Nicola Hall, Linda Birt, Fiona M Walter

**Affiliations:** The Primary Care Unit, Department of Public Health and Primary Care, University of Cambridge, Cambridge, CB1 8SR UK; Evaluation, Research and Development Unit, School of Medicine, Pharmacy and Health, Durham University, Durham, TS17 6BH UK; General Practice and Primary Care Academic Centre, University of Melbourne, Carlton, VIC 3053 Australia; GP & Clinician Scientist, The Primary Care Unit, Department of Public Health & Primary Care, University of Cambridge, Cambridge, CB1 8SR UK

**Keywords:** Calendar, Landmarks, Retrospective, Recall, Cancer, Symptoms

## Abstract

**Background:**

Late diagnosis is considered to be a major factor contributing to poorer cancer survival rates in the UK. Interventions have focussed on the promotion of earlier diagnosis in patients with potential cancer symptoms. However, to assess the effectiveness of these interventions, the time from symptom onset to presentation needs to be reliably and accurately measured. This qualitative study explored the use of calendar landmarking instruments in cancer symptom research.

**Methods:**

We performed a secondary analysis of transcripts of interviews using the calendar landmarking instrument, undertaken with patients who had either been diagnosed with cancer (n = 40, IRCO study, Western Australia), or who had symptoms suggestive of cancer (n = 38, SYMPTOM study, North East and Eastern England). We used constant comparison methods to identify use of the calendar landmarking instruments and the impact of their application.

**Results:**

The calendar landmarking instrument appeared to help many patients, either by acting as a prompt or helping to refine recall of events. A combination of personal (e.g. birthday) and national (e.g. Christmas) landmarks seemed to be the most effective. Calendar landmarking instruments appeared more useful where the time period between onset of symptoms and date of first consultation was less than three months. The interviewee’s age, gender and cancer type did not appear to influence whether or not the instrument facilitated recall, and there were no instances where the use of the instrument resulted in the disclosure of a new first symptom. Symptoms of similar chronic conditions could create difficulties when applying the instrument; it was difficult for these participants to characterise and disentangle their symptoms which prompted their decisions to seek help. Some participants tended to prefer to use their own, already personalised, diaries to assist in their recall of events.

**Conclusions:**

This study is the first to describe the potential role of calendar landmarking instruments to support research interviews which explore symptoms and events along the cancer diagnostic pathway. The major challenge remains as to whether they actually improve accuracy of recall.

## Background

Late diagnosis is considered to be a major factor contributing to poorer cancer survival rates. Recent research has focussed on interventions to promote earlier diagnosis in people with symptoms suggestive of cancer [1]. In order to assess the effectiveness of these interventions the time from symptom onset to diagnosis and onset of treatment needs to be reliably and accurately measured [2]. Time to diagnosis encompasses the ‘patient interval’ from first noticing a symptom, via symptom appraisal and help-seeking decisions to first presentation in primary care (also known as ‘time to presentation’), as well as the ‘healthcare professional interval’ from first presentation via investigations to diagnosis and initiation of treatment [3,4]. The Aarhus Statement emphasises the importance of accurate definitions and measurement of these time intervals by clinicians and researchers [5]. This type of research often involves retrospective recall of symptom onset by patients; such recall is subject to many types of error which may affect the accuracy, reliability and completeness of data collection.

Calendar landmarking instruments have been widely used across many research fields to increase the reliability of dating, and to improve consistency and completeness of data [6]. A number of different calendar landmarking instruments have been devised, all of which aim to enhance recall of autobiographical memories through the use of significant private or public events as ‘landmarks’. Using landmark events can also enhance sequencing of events which reduces the risk of events being omitted. Calendar landmarking instruments usually incorporate a visual display of the time period in question and may gather information about one or more life domains as required. Calendar landmarking instruments were initially used in social sciences research. For example, they were shown to improve the accuracy of recall of living arrangements, involvement in education, employment and financial status over a period of months to years [7]. Calendar landmarking instruments have also been used in relation to health behaviours. The Timeline Follow Back method was shown to have a slightly higher reliability and sensitivity than traditional quantity-frequency measurements of alcohol consumption [8], and women’s recall of their contraceptive use showed good agreement between a calendar landmarking instrument and medical records [9].

However, calendar landmarking instruments have seldom been used while collecting data for analysing time to clinical diagnosis for any conditions including cancer. The only example is from Corner and colleagues who investigated patients’ recollections of symptoms before a diagnosis of lung cancer using a timeline called “What Happened to Me?” to assist recall of events dating back from the time of diagnosis to the onset of the first symptoms. Patients were also asked to recall life events that had occurred at the same time, and their recollections were found to agree with their primary care and hospital records [10,11]. Despite this limited evidence, the Aarhus Statement recommends the use of calendar landmarking techniques in studies of cancer diagnostic intervals. This paper reports a qualitative evaluation of our experiences using calendar landmarking methods in two studies of symptomatic cancer diagnosis.

## Methods

### The datasets

The datasets were obtained from one completed study set in Australia and one on-going study set in England. Both studies involved in-depth interviews with patients who had either been diagnosed with cancer (Australia) or who had symptoms of possible cancer (England). The Australian study, set in Western Australia, aimed to identify patient, doctor and health factors that affect time to diagnosis and treatment outcomes in rural patients with cancer of the prostate, breast, colorectum or lung [12,13]. Face-to-face in-depth interviews were undertaken between 2009 and 2010 by 5 interviewers. In total 66 interviews were undertaken, but 26 involved people with screen-detected cancer therefore calendar landmarking instruments were not used. All remaining 40 interviews were included in this secondary analysis.

The English study, set in the East and the North East of England, is a cohort study with a nested qualitative component, aiming to characterise factors affecting symptom appraisal and associations with cancer in people referred with symptoms suspicious of lung, colorectal or pancreatic cancer. These face-to-face in-depth interviews were undertaken between January and August 2011 (n = 38), by one experienced qualitative researcher in each region. The primary analysis is in progress.

While interviews with patients after cancer diagnosis may be subject to recall bias, recruitment of patients earlier in the diagnostic pathway will inevitably include people who go on to have both cancer and other diagnoses. We therefore chose to combine the datasets in order to enrich our understanding of patient recollections of the onset and appraisal of their symptoms, and the decisional factors leading to help-seeking in both patient groups. In both sets of interviews the participants were asked about the date of onset of the first symptom and the date of first consultation with a health care professional, using a calendar landmarking instrument to aid recall of their pathways towards diagnosis and the start of treatment. All interviews were audio-taped and professionally transcribed.

Ethical approval was obtained for each study. This was gained from the University of Western Australia (UWA) Human Research Ethics Committee (RA/4/1/2242) and the Cambridgeshire 3 Research Ethics Committee (10/H0306/50). Written informed consent was obtained from each participant prior to start of the interview.

### The calendar landmarking instruments

**Figure 1:**
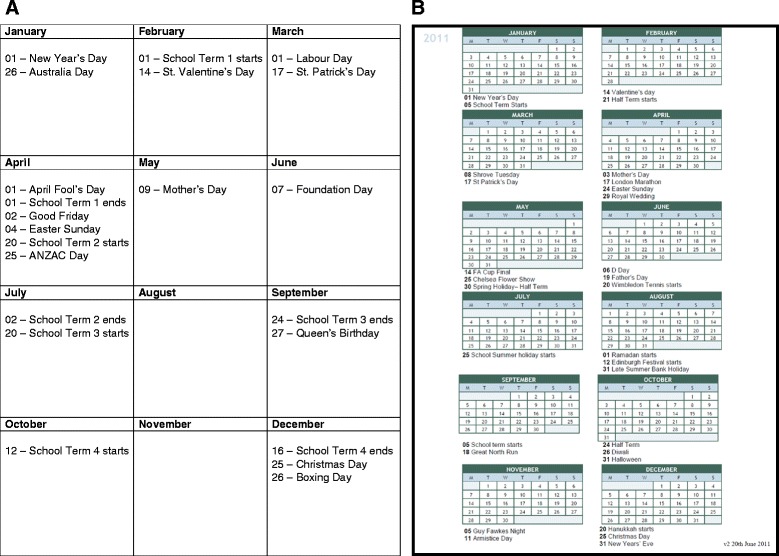
**Calendar Landmarking Instruments. A** Calendar Landmarking Instrument- Australia (CLI-Au). **B** Calendar Landmarking Instrument- UK (CLI-UK).

The Australian interviewers used a range of strategies to try to improve accuracy of recall, including a calendar landmarking instrument (CLI-Au) and an ordinary calendar. The CLI-Au consisted of a list of dates, ordered by the months of the year, including international dates such as Christmas and New Year; national dates such as Australia Day and public holidays; local dates such as festivals; and sporting dates such as the football World Cup, Olympic Games and Melbourne Cup, see Figure 1A. The interviewers were asked to encourage the respondents to think of their own personal ‘landmarks’ such as holidays, birthdays or wedding anniversaries, which could be added to the CLI-Au. The interviewers were asked to use these landmark events to identify significant events including the onset of symptoms and the first appointment with their general practitioner (GP).

A calendar landmarking instrument was specifically developed for the English study (CLI-UK), and the interviewers were instructed to use similar strategies to their Australian colleagues. The CLI-UK included similar international dates, with the national and local landmarks modified to reflect UK, rather than Australian events, see Figure 1B.

### Analysis

The analysis was undertaken using constant comparison techniques involving reading and re-reading of transcripts and comparison between transcripts to identify use of the calendar landmarking instruments and the impact of their application [14]. Two authors (KM, CC) read all the transcripts; more than half of the transcripts were also read by at least two of the other researchers so that the analysis could be interpreted and refined though regular discussions. Once evidence of use of the calendar landmarking instruments had been established, all dates elicited for symptom onset and initial consultation in the first part of the interview (before the instruments were used) were compared with those elicited following use of the instruments by the interviewers. Examples of where the instruments aided the respondent’s recall were identified, as well as examples where the calendar landmarking instrument did not help or tighten recall of dates. We also assessed which landmarks had been most helpful and which had been less useful.

## Results

### Participant characteristics

Transcripts from 78 interviews were included (40 Australia; 38 England). Table 1 shows the characteristics of the interviewees in Australia and England, including their diagnoses.

**Table 1:** **Participant Characteristics**

	**Australian set**	**English set**
	**(n = 40)**	**(n = 38)**
**Age**		
<50	9	5
>50	31	33
**Gender**		
Female	25	17
Male	15	21
**Ethnicity**		
White	39	37
Other	1^a^	1^b^
**Cancer site** ^c^		
Breast	16 C	-
Lung	5 C	4C, 13 N
Colorectal	14 C	18 N
Prostate	5 C	-
Pancreas	-	2C, 1 N

^a^Aboriginal.

^b^Black African.

C = diagnosed with cancer; N = not diagnosed with cancer.

### Usefulness of calendar landmarking instruments

In more than half of the interviews the calendar landmarking instrument appeared to help participants recall the date of their first symptom onset and/or the date of the first GP consultation. It was useful in two key ways. First, interviewees used the calendar landmarking instrument as a prompt to their recall of events that had happened some months prior to their initial estimate of time of symptom onset. They used personal landmarks to pinpoint a time when a particular symptom had started, and often the interviewer’s role was mainly as facilitator – to listen and prompt further information as required.

*I: So your first symptom was your shoulder pain, right shoulder pain, and that was in October/November ‘08?*

R’s wife: It was probably there before that too ….

*I: So maybe we’re looking at … just to pinpoint that first initial symptom…*

R’s wife: Yes, so about May that year. No, you were driving the motor home at one stage…, we were going down for the kids’ birthdays in Bunbury and you said ‘oh, me shoulder’s annoying me’ and because it’s fairly heavy to drive and I know when I drive it I get sore shoulders, and whether that was the beginnings of it or not, that was February of that year. Yeah, ‘cause we used to go down there for the kids’ birthdays, ‘cause two of our grandchildren have birthdays a day apart down there.

*I: So what days are those birthdays?*

R’s wife: Eighteenth and 19th of February. …yeah but that sort of subsided and didn’t matter. We thought, oh well it was because he was driving. And of course that was way before….

*[Australian patient, lung cancer]*

Second, the interviewer used events from the calendar landmarking instrument to establish the date of first symptom onset. This could help interviewees refine a vague recollection such as ‘about summer last year’ to a specific range of days relating to a recalled event. The recalled event could be a national or international one:

R1: When did I go to the doctors? Two months ago?

*I: Before Christmas then?*

R2: Yeah, two or three months ago it was, but it was happening before that, long before that… So it was about summer last year.. when it started, yes.

*I: Okay. So would that be before the August public holiday?*

R2: It was before that, it was before August, it was during the kids’ school holidays…..

*I: So was that before July? Before they went on holiday?*

R1: Let’s say it happened at the World Cup finals.

*I: World Cup, around there was it? The World Cup started in June.*

R2: That would be about right because during the World Cup he was up and down going to the toilet and he missed part of the football, if you remember rightly.

R1: You have a better memory than I have pet!

*[English patient, suspected colorectal cancer]*

The recalled event could also be one of personal significance:

*I: So, around May or June, were there any events… that would be easy to remember?*

R: Yes I, in fact I would say that it’s specifically June… because my daughter was admitted in April.. and was discharged on the same date.. that was this last.. you know.. Prime Minister debate thing.

*I: Oh the General Election…*

R: Yeah for the General Election.

*I: So I think that was on the 6th of May.*

R: Okay, 6th of May, yes. Then I think it was a week or two or three weeks after that that the Social Service lady visited us.. yeah it was around mid-May that [the] arrangement was made for me to be going to the charity to collect.. the food stuff…

*I: And.. did the cold [and fever] start after that?*

R: Yeah, in fact I can remember, it was one of the days that I was going to collect [the] food pack, ..that day in fact was the day I started feeling cold. Yes, yes, so I can’t tell you this specifically but normally it was on a Thursday that I used to go and collect that thing from the charity…..

[English patient, suspected lung cancer]

There were no instances where the use of the calendar landmarking instrument was actively unhelpful, resulting in either interviewer or respondent frustration or even termination of the interview. There were, however, examples of where the calendar landmarking instrument did not appear to help recall, see Table 2.

**Table 2:** **Examples where calendar landmarking instrument did not facilitate recall**

**Participant**	**Interviewee comments**
Australian patient, colorectal cancer	I: We’ve got some tools here to maybe help you remember. Was this last year?
	R: Yep.
	I: So that’s 2008. And we’ve got some events on this calendar that might help you remember when those symptoms first started. Was it winter or summer can you remember that sort of thing…
	R: You really don’t know how bad my memory is, do ya … um….I’ve really got no idea.
	I: Got no idea … birthdays … race rounds? Just roughly, when those symptoms started, [when] the bleeding first started.
	R: I’ve got no idea. Honestly, no idea. Nuh.
Australian patient, breast cancer	I: Hopefully this will all make it a bit easier. We’ve got some dates down on the calendar there which uh, the idea is to try and help you remember what you were doing around these times…
	R: I’ve got no idea. Five days, yeah… my days blend in…I run my own business, I’ve got two teenaged girls….and you can forget it.
English patient, suspected lung cancer	I: Do you remember exactly when it was when you went to see the doctor…?
	R: No.
	I: Was it before Christmas?
	R: Oh yes.
	I: I have here, it’s … a tool to help people remember, I don’t know how helpful it’s going to be. (Laughs) So it was before Christmas, was it after bonfire night, November was it?
	R: Yes.
	I: After bonfire night… after Armistice Day… no?
	R: I’m trying to think… It’s got to be November… I’m sorry, I really can’t remember.
English patient, suspected lung cancer	I: So, do you think that was before Easter?
	R: When would it be? It’s not so long ago, is it?
	I: Was it before the Royal wedding?
	R: Well you see this… these are the letters for the whole, when I was going to get… now that one was the 10th May.
	I: So is that before…?
	R: So it would have been before that, yeah.. Yes, so from… it would be the back end of um, it would be the back end of ah, ah, back end of um, April.
	I: Back end of April. So was that around all the public holidays and the Royal wedding and Easter? Does that ring a bell now that time?
	R: No, that’s the one…that’s the last [xray] that got took, that one.

### Factors affecting usefulness of calendar landmarking instruments

The time since symptom onset appeared to affect the usefulness of the calendar landmarking instruments in that they were used more often and added to the precision of dates where the time period between onset of symptoms and date of first consultation was less than three months. In contrast, the interviewee’s age, gender or cancer type did not appear to influence whether or not the calendar landmarking instrument facilitated recall; there were no instances where the use of the calendar landmarking instrument resulted in the disclosure of a new first symptom.

International events such as Christmas and New Year, and the public holidays in England, were the most useful ‘landmarks’, and personal events identified by interviewee, particularly family birthdays or holidays, were also frequently mentioned. Sporting events had some success as landmarks, including the international Olympic Games as well as a local horse race meeting in Australia. Unique events were also successful as landmarks: most noteworthy was the Royal Wedding in April 2011, and there were two interviewees who had been symptomatic for a while and who said the 2010 UK General Election was a helpful landmark. Interestingly, landmarks such as World War 2 anniversaries did not seem helpful for either the older or younger interviewees.

Where participants had existing chronic conditions, specifically where symptoms were of a similar nature such as chronic obstructive pulmonary disease and lung cancer, or benign prostatic hypertrophy and prostate cancer, we found that this could create difficulties when applying the instrument. It was difficult for these participants to characterise and disentangle their symptoms which prompted their decisions to seek help. During the interviews some participants had access to their own diary; in these instances they tended to prefer to use this rather than the calendar landmarking instrument to assist in their recall of events because they were so personalised already.

## Discussion

### Principal findings

This study is the first to describe the potential role of calendar landmarking instruments to support research interviews which explore symptoms and events along the cancer diagnostic pathway. While they were not useful in all interviews, this qualitative exploration suggests that using calendars, particularly involving the use of personal (birthdays, holidays) and international (public holidays, Christmas) events, may facilitate recall. Using the landmarks often contributed to the refinement of the date of first symptom onset, and discussions of this time period appeared to enable some participants to re-visit their symptom appraisal and clarify the decisional factors which led them to seek healthcare. Greater insights were therefore gained into the patient processes and contributing factors which influenced symptom appraisal and seeking healthcare with the use of this visual presentation of time.

### Comparison with existing literature

Our study findings are consistent with the results of a review of calendar landmarking instruments which describes how they can be used to discuss specific periods of time and explore sensitive issues with participants [6]. Using a calendar instrument may make it easier to discuss sensitive issues for both the researchers and the respondents where referring to information written on the instrument can help with the discussion [15]. Although we found that some people with similar chronic conditions could find it difficult to characterise and disentangle their symptoms, others found that calendar landmarking could be of particular value when their symptoms became more persistent or severe, or were of an episodic or vague nature [16].

In both our primary studies the calendar landmarking instruments were implemented by the researchers during the interviews and were used by the researcher to stimulate discussion. This contrasts with a Dutch study where the participants were sent the instrument to self-complete during telephone interviews [7]. Both implementation techniques were found to assist in retrospective recall of the participants’ significant events, and we found that applying the instrument during the interview ensured the flow was un-interrupted. It was interesting that in our study some participants found that using their own diary was of more use than the calendar landmarking instrument, because it was already so personal. Future research may explore personal preferences for this as an alternative to standardised calendar landmarking instruments.

### Strengths and limitations

This was a qualitative study involving secondary analysis of transcripts of interviews where a number of researchers had applied a calendar landmarking method to support patient recall of events prior to a cancer diagnosis. It involved 78 interviews across two countries and therefore reflects a broad experience of their use. It was not designed to assess the impact of this method to improve accuracy of recall. This would be a very challenging objective given the absence of any true reference dates.

There were seven interviewers who each used the calendar landmarking instruments in slightly different ways. We had initially envisaged a more formal introduction of the instruments at a set point during the interviews, with respondents being invited to personalise the landmarking tool with the interviewer before starting any discussion about dates of symptoms or GP consultations. In practice, however, the calendar landmarking instruments were used in a much more pragmatic and sensitive manner by each interviewer, and introduced when they felt it was most appropriate for the interviewee.

### Implications for research and practice

Early diagnosis cancer research has been characterised by its complexity, a lack of transparency in multi-disciplinary perspectives, and a poorly developed set of definitions and methodological tools [2]. The Aarhus Statement has suggested that one approach to improve the quality of studies of diagnostic intervals in symptomatic cancer is the use of calendar landmarking instruments. This study describes different ways in which using this technique may help participants to recall events leading to their cancer diagnosis, although we acknowledge that calendars were most use for those with shorter times from first symptoms to consultation. Despite being a challenging objective, further research on the usefulness of calendar landmarking instruments could include validation against reports from relatives, friends and the patient’s wider social group including workplace, and GP records. Further testing could include inter- and intra-person reliability measures to compare the use of calendar-landmarking instruments with no use. Further research is also required to establish whether calendar landmarking instruments could also have value in early diagnosis research across other diseases and conditions.

## Conclusions

In conclusion, this qualitative exploration is the first to describe the potential role of calendar landmarking instruments to support research interviews which explore symptoms and events along the cancer diagnostic pathway. The major challenge remains as to whether they actually improve accuracy of recall.
